# 3,8-Bis(4-chloro­phen­yl)-4,7-dimethyl­tricyclo­[4.2.2.0^2,5^]deca-3,7-diene

**DOI:** 10.1107/S1600536808003206

**Published:** 2008-02-06

**Authors:** Masaaki Tomura, Yoshiro Yamashita

**Affiliations:** a Institute for Molecular Science, Myodaiji, Okazaki 444-8585, Japan; b Department of Electronic Chemistry, Interdisciplinary Graduate School of Science and Engineering, Tokyo Institute of Technology, Nagatsuta, Midori-ku, Yokohama 226-8502, Japan

## Abstract

The title tricyclic diene, C_24_H_22_Cl_2_, is the product of thermal ring-opening of a corresponding basketane (penta­cyclo­[4.4.0.0^2,5^.0^3,8^.0^4,7^]deca­ne) derivative. The cyclo­butene ring is planar to within 0.0032 (12) Å and its geometry is normal. The two 4-chloro­phenyl groups are oriented in an approximately face-to-face conformation with a dihedral angle of 44.14 (6)° between them. The 4-chloro­phenyl group bonded to the cyclo­butene ring lies almost in the plane of the cyclo­butene ring, with a dihedral angle of 8.29 (17)° between the ring planes. The average intra­molecular C⋯C distance between the two C=C bonds is 2.92 Å. In the crystal structure, the mol­ecules are well separated with no close C—H⋯Cl or C—H⋯π inter­molecular inter­actions.

## Related literature

For the preparation of the title compound, see: Tezuka *et al.* (1976 ▶); Mukai *et al.* (1981 ▶). For cage compounds, see: Osawa & Yonemitsu (1992 ▶). For the crystal structures of compounds with a tricyclo­[4.2.2.0^2,5^]deca-3,7-diene skeleton, see: Lemley *et al.* (1976 ▶); Hanson (1981 ▶); Mehta *et al.* (1990 ▶, 2003 ▶). For related literature, see: Allen (1984 ▶, 2002 ▶); Allen *et al.* (1987 ▶).
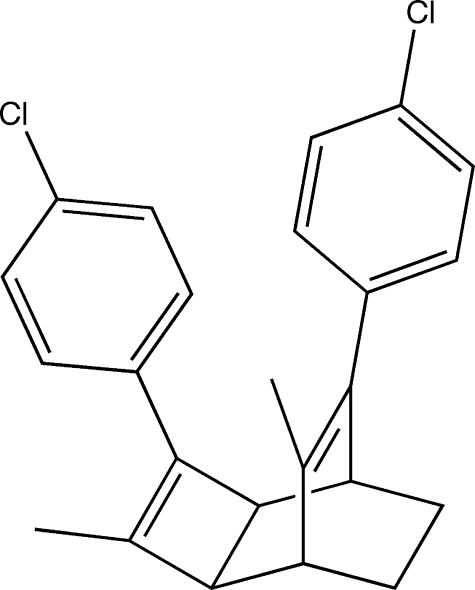


## Experimental

### 

#### Crystal data

C_24_H_22_Cl_2_
*M* *_r_* = 381.32
Monoclinic, 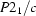

*a* = 8.3389 (7) Å
*b* = 21.2224 (12) Å
*c* = 11.6074 (13) Å
β = 103.732 (7)°
*V* = 1995.5 (3) Å^3^
*Z* = 4
Cu *K*α radiation
μ = 2.94 mm^−1^
*T* = 295 (1) K
0.48 × 0.35 × 0.25 mm

#### Data collection

Enraf–Nonius CAD-4 diffractometer
Absorption correction: ψ scan (North *et al.*, 1968 ▶) *T*_min_ = 0.328, *T*_max_ = 0.484
4348 measured reflections
4070 independent reflections
2872 reflections with *I* > 2σ(*I*)
*R*_int_ = 0.021
3 standard reflections frequency: 120 min intensity decay: 1.3%

#### Refinement

*R*[*F* ^2^ > 2σ(*F* ^2^)] = 0.050
*wR*(*F* ^2^) = 0.160
*S* = 1.04
4070 reflections
237 parameters
H-atom parameters constrained
Δρ_max_ = 0.20 e Å^−3^
Δρ_min_ = −0.33 e Å^−3^

### 

Data collection: *CAD-4 EXPRESS* (Enraf–Nonius, 1992 ▶); cell refinement: *CAD-4 EXPRESS*; data reduction: *TEXSAN* (Rigaku/MSC, 2000 ▶); program(s) used to solve structure: *SHELXS97* (Sheldrick, 2008 ▶); program(s) used to refine structure: *SHELXL97* (Sheldrick, 2008 ▶); molecular graphics: *PLATON* (Spek, 2003 ▶); software used to prepare material for publication: *SHELXL97*.

## Supplementary Material

Crystal structure: contains datablocks global, I. DOI: 10.1107/S1600536808003206/sj2459sup1.cif

Structure factors: contains datablocks I. DOI: 10.1107/S1600536808003206/sj2459Isup2.hkl

Additional supplementary materials:  crystallographic information; 3D view; checkCIF report

## Figures and Tables

**Fig. 1.**
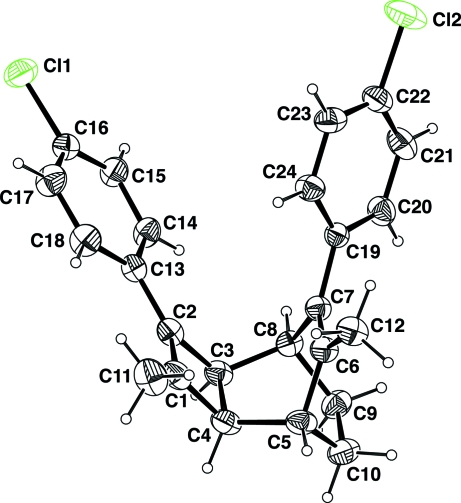
The molecular structure of (I), with atom labels and 20% probability displacement ellipsoids for non-H atoms. H atoms are shown as small spheres with arbitrary radii.

## supplementary crystallographic information

### Comment

There is considerable interest in the chemistry of highly strained polycyclic "cage" compounds (Osawa & Yonemitsu, 1992). The title tricyclic diene, (I), Fig 1, 3,8-bis(4-chlorophenyl)-4,7-dimethyltricyclo[4.2.2.0^2,5^] deca-3,7-diene, C_24_H_22_Cl_2_, is the product of thermal ring-opening of a corresponding basketane (pentacyclo[4.4.0.0^2,5^.0^3,8^.0^4,7^]decane) and reverts to the basketane derivative quantitatively upon irradiation (Tezuka *et al.*, 1976). A search for tricyclo[4.2.2.0^2,5^]deca-3,7-diene skeleton in the Cambridge Structural Database (Version 5.29; Allen, 2002) gave only four examples, CTCYDD (Lemley *et al.*, 1976), CNUNDC (Hanson, 1981), KEVGEX (Mehta *et al.*, 1990) and GACFIA (Mehta *et al.*, 2003).

Bond lengths and angles in the molecule are within the normal ranges (Allen *et al.*, 1987) and the geometry of the cyclobutene ring is also similar to that of cyclobutene (Allen, 1984). The cyclobutene ring adopts a planar, rather than a puckered conformation, where the maximum deviation of the fitted atoms from the least-squares plane is 0.0032 (12) Å. The C3—C4 bond distance [1.562 (3) Å] in the cyclobutene ring is 1.8% shorter than the corresponding distance in the 2,3,4,5-tetrachloro derivative (Lemley *et al.*, 1976). The two 4-chlorophenyl groups are oriented in an approximately face-to-face conformation with a dihedral angle of 44.14 (6)° between them. The 4-chlorophenyl group bonded to the cyclobutene ring lies almost in the plane of the cyclobutene ring with a dihedral angle of 8.29 (17)° between the ring planes. Upon irradiation, an intramolecular photocyclization occurs between the C1?C2 and C6?C7 bonds. Intramolecular C1···C6 and C2···C7 distances are 2.918 (3) and 2.921 (3) Å, respectively, and the dihedral angle between the cyclobutene plane (C1–C4) and the C5–C8 plane is 60.89 (12)°. In the crystal structure the molecules are well separated with no close C—H···Cl or C—H···π intermolecular interactions.

### Experimental

The compound (I) was synthesized according to a literature method (Tezuka *et al.*, 1976; Mukai *et al.*, 1981). Colorless crystals of (I) suitable for X-ray analysis were grown from a dichloromethane solution.

### Refinement

All H atoms were placed in geometrically calculated positions and refined using a riding model, with C—H distances of 0.93, 0.96, 0.97 and 0.98 Å for aromatic, methyl, methylene and methine H atoms, respectively, and with *U*_iso_(H) = 1.2*U*_eq_(C) for aromatic, methylene and methine or 1.5*U*_eq_(C) for methyl H atoms.

### Crystal data

**Table:**

C_24_H_22_Cl_2_	*F*_000_ = 800
*M**_r_* = 381.32	*D*_x_ = 1.269 Mg m^−^^3^
Monoclinic, *P*2_1_/*c*	Cu *K*α radiation λ = 1.54178 Å
Hall symbol: -P 2ybc	Cell parameters from 25 reflections
*a* = 8.3389 (7) Å	θ = 22.7–42.6º
*b* = 21.2224 (12) Å	µ = 2.94 mm^−^^1^
*c* = 11.6074 (13) Å	*T* = 295 (1) K
β = 103.732 (7)º	Block, colorless
*V* = 1995.5 (3) Å^3^	0.48 × 0.35 × 0.25 mm
*Z* = 4	

### Data collection

**Table:**

Enraf–Nonius CAD-4 diffractometer	*R*_int_ = 0.021
Monochromator: graphite	θ_max_ = 74.2º
*T* = 295(1) K	θ_min_ = 4.2º
ω–2θ scans	*h* = 0→10
Absorption correction: ψ scan(North *et al.*, 1968)	*k* = 0→26
*T*_min_ = 0.328, *T*_max_ = 0.484	*l* = −14→14
4348 measured reflections	3 standard reflections
4070 independent reflections	every 120 min
2872 reflections with *I* > 2σ(*I*)	intensity decay: 1.3%

### Refinement

**Table:**

Refinement on *F*^2^	Secondary atom site location: difference Fourier map
Least-squares matrix: full	Hydrogen site location: inferred from neighbouring sites
*R*[*F*^2^ > 2σ(*F*^2^)] = 0.050	H-atom parameters constrained
*wR*(*F*^2^) = 0.160	*w* = 1/[σ^2^(*F*_o_^2^) + (0.0822*P*)^2^ + 0.357*P*] where *P* = (*F*_o_^2^ + 2*F*_c_^2^)/3
*S* = 1.04	(Δ/σ)_max_ = 0.007
4070 reflections	Δρ_max_ = 0.20 e Å^−^^3^
237 parameters	Δρ_min_ = −0.32 e Å^−^^3^
Primary atom site location: structure-invariant direct methods	Extinction correction: none

### Special details

**Table:**

Geometry. All e.s.d.'s (except the e.s.d. in the dihedral angle between two l.s. planes) are estimated using the full covariance matrix. The cell e.s.d.'s are taken into account individually in the estimation of e.s.d.'s in distances, angles and torsion angles; correlations between e.s.d.'s in cell parameters are only used when they are defined by crystal symmetry. An approximate (isotropic) treatment of cell e.s.d.'s is used for estimating e.s.d.'s involving l.s. planes.
Refinement. Refinement of *F*^2^ against ALL reflections. The weighted *R*-factor *wR* and goodness of fit *S* are based on *F*^2^, conventional *R*-factors *R* are based on *F*, with *F* set to zero for negative *F*^2^. The threshold expression of *F*^2^ > σ(*F*^2^) is used only for calculating *R*-factors(gt) *etc*. and is not relevant to the choice of reflections for refinement. *R*-factors based on *F*^2^ are statistically about twice as large as those based on *F*, and *R*- factors based on ALL data will be even larger.

### Fractional atomic coordinates and isotropic or equivalent isotropic displacement parameters (Å^2^)

**Table:**

	*x*	*y*	*z*	*U*_iso_*/*U*_eq_	
Cl1	0.49013 (12)	0.19930 (4)	1.21790 (6)	0.1028 (3)	
Cl2	1.19192 (12)	0.35095 (4)	0.98762 (11)	0.1347 (4)	
C1	0.7177 (3)	0.00447 (11)	0.7920 (3)	0.0761 (7)	
C2	0.6407 (3)	0.05850 (10)	0.8097 (2)	0.0661 (5)	
C3	0.5989 (3)	0.07597 (11)	0.6796 (2)	0.0652 (5)	
H3	0.4802	0.0750	0.6432	0.078*	
C4	0.6896 (3)	0.01375 (11)	0.6609 (2)	0.0749 (7)	
H4	0.6163	−0.0179	0.6144	0.090*	
C5	0.8386 (3)	0.03011 (12)	0.6096 (2)	0.0753 (7)	
H5	0.9014	−0.0079	0.6011	0.090*	
C6	0.9467 (3)	0.07722 (11)	0.6897 (2)	0.0656 (5)	
C7	0.8686 (2)	0.13047 (10)	0.70463 (19)	0.0600 (5)	
C8	0.6880 (2)	0.13196 (11)	0.63804 (19)	0.0618 (5)	
H8	0.6364	0.1719	0.6515	0.074*	
C9	0.6785 (3)	0.12218 (14)	0.5049 (2)	0.0793 (7)	
H9A	0.5639	0.1196	0.4614	0.095*	
H9B	0.7290	0.1576	0.4743	0.095*	
C10	0.7692 (3)	0.06101 (14)	0.4880 (2)	0.0858 (8)	
H10A	0.8585	0.0704	0.4503	0.103*	
H10B	0.6934	0.0323	0.4372	0.103*	
C11	0.8109 (4)	−0.04637 (13)	0.8691 (3)	0.1037 (10)	
H11A	0.9228	−0.0472	0.8609	0.156*	
H11B	0.7596	−0.0863	0.8455	0.156*	
H11C	0.8102	−0.0382	0.9503	0.156*	
C12	1.1250 (3)	0.06200 (12)	0.7406 (3)	0.0787 (7)	
H12A	1.1803	0.0985	0.7801	0.118*	
H12B	1.1756	0.0497	0.6779	0.118*	
H12C	1.1333	0.0281	0.7964	0.118*	
C13	0.6085 (3)	0.09224 (10)	0.9117 (2)	0.0617 (5)	
C14	0.5426 (3)	0.15260 (11)	0.8976 (2)	0.0721 (6)	
H14	0.5194	0.1709	0.8227	0.086*	
C15	0.5102 (3)	0.18636 (12)	0.9912 (2)	0.0759 (6)	
H15	0.4683	0.2271	0.9798	0.091*	
C16	0.5404 (3)	0.15904 (12)	1.1008 (2)	0.0710 (6)	
C17	0.6047 (4)	0.09927 (14)	1.1188 (2)	0.0875 (8)	
H17	0.6248	0.0809	1.1936	0.105*	
C18	0.6390 (4)	0.06678 (12)	1.0248 (2)	0.0838 (7)	
H18	0.6840	0.0266	1.0375	0.101*	
C19	0.9420 (3)	0.18508 (10)	0.77687 (19)	0.0574 (5)	
C20	0.9320 (3)	0.24474 (12)	0.7278 (2)	0.0772 (7)	
H20	0.8739	0.2505	0.6496	0.093*	
C21	1.0060 (4)	0.29581 (13)	0.7920 (3)	0.0924 (9)	
H21	0.9986	0.3355	0.7573	0.111*	
C22	1.0901 (3)	0.28762 (13)	0.9069 (3)	0.0810 (7)	
C23	1.0954 (3)	0.23022 (14)	0.9607 (2)	0.0826 (7)	
H23	1.1486	0.2256	1.0403	0.099*	
C24	1.0210 (3)	0.17907 (12)	0.8956 (2)	0.0736 (6)	
H24	1.0239	0.1400	0.9321	0.088*	

### Atomic displacement parameters (Å^2^)

**Table:**

	*U*^11^	*U*^22^	*U*^33^	*U*^12^	*U*^13^	*U*^23^
Cl1	0.1386 (7)	0.1011 (5)	0.0714 (4)	−0.0031 (5)	0.0303 (4)	−0.0133 (3)
Cl2	0.1042 (6)	0.0980 (6)	0.1974 (11)	−0.0306 (5)	0.0271 (6)	−0.0685 (6)
C1	0.0663 (14)	0.0549 (12)	0.1071 (19)	0.0000 (10)	0.0209 (13)	−0.0022 (12)
C2	0.0562 (12)	0.0574 (12)	0.0851 (15)	0.0019 (9)	0.0176 (11)	0.0013 (11)
C3	0.0487 (10)	0.0662 (12)	0.0783 (14)	0.0005 (9)	0.0103 (10)	−0.0110 (11)
C4	0.0571 (12)	0.0666 (14)	0.1002 (18)	−0.0060 (10)	0.0171 (12)	−0.0246 (13)
C5	0.0591 (12)	0.0737 (14)	0.0931 (17)	0.0031 (11)	0.0181 (12)	−0.0285 (13)
C6	0.0520 (11)	0.0691 (13)	0.0746 (14)	0.0025 (10)	0.0127 (10)	−0.0114 (11)
C7	0.0524 (11)	0.0636 (12)	0.0625 (12)	0.0005 (9)	0.0110 (9)	−0.0040 (9)
C8	0.0530 (11)	0.0680 (12)	0.0626 (12)	0.0069 (9)	0.0104 (9)	−0.0029 (10)
C9	0.0639 (13)	0.108 (2)	0.0635 (13)	0.0046 (13)	0.0109 (11)	−0.0095 (13)
C10	0.0675 (14)	0.113 (2)	0.0764 (16)	−0.0002 (14)	0.0156 (12)	−0.0309 (15)
C11	0.101 (2)	0.0642 (15)	0.150 (3)	0.0211 (14)	0.039 (2)	0.0158 (17)
C12	0.0565 (12)	0.0775 (15)	0.0983 (18)	0.0090 (11)	0.0107 (12)	−0.0098 (13)
C13	0.0554 (11)	0.0564 (11)	0.0729 (13)	0.0017 (9)	0.0141 (10)	0.0067 (10)
C14	0.0868 (16)	0.0643 (13)	0.0687 (13)	0.0126 (11)	0.0255 (12)	0.0139 (11)
C15	0.0925 (17)	0.0610 (13)	0.0795 (15)	0.0102 (12)	0.0308 (13)	0.0075 (11)
C16	0.0742 (14)	0.0717 (14)	0.0666 (13)	−0.0071 (11)	0.0157 (11)	−0.0029 (11)
C17	0.105 (2)	0.0858 (17)	0.0691 (15)	0.0102 (16)	0.0156 (14)	0.0184 (13)
C18	0.0937 (18)	0.0691 (15)	0.0869 (17)	0.0185 (13)	0.0180 (14)	0.0162 (13)
C19	0.0526 (10)	0.0575 (11)	0.0636 (12)	0.0002 (8)	0.0163 (9)	−0.0031 (9)
C20	0.0820 (16)	0.0708 (15)	0.0762 (15)	−0.0063 (12)	0.0137 (12)	0.0103 (12)
C21	0.095 (2)	0.0620 (14)	0.121 (2)	−0.0147 (13)	0.0270 (18)	0.0067 (15)
C22	0.0630 (14)	0.0708 (15)	0.112 (2)	−0.0092 (11)	0.0267 (14)	−0.0259 (15)
C23	0.0796 (17)	0.0903 (18)	0.0723 (15)	−0.0016 (13)	0.0067 (12)	−0.0191 (14)
C24	0.0837 (16)	0.0633 (13)	0.0685 (14)	−0.0029 (11)	0.0075 (12)	0.0006 (11)

### Geometric parameters (Å, °)

**Table:**

Cl1—C16	1.739 (3)	C11—H11A	0.9600
Cl2—C22	1.740 (3)	C11—H11B	0.9600
C1—C2	1.353 (3)	C11—H11C	0.9600
C1—C11	1.496 (4)	C12—H12A	0.9600
C1—C4	1.496 (4)	C12—H12B	0.9600
C2—C13	1.462 (3)	C12—H12C	0.9600
C2—C3	1.513 (3)	C13—C18	1.386 (3)
C3—C8	1.538 (3)	C13—C14	1.388 (3)
C3—C4	1.562 (3)	C14—C15	1.381 (3)
C3—H3	0.9800	C14—H14	0.9300
C4—C5	1.540 (3)	C15—C16	1.366 (3)
C4—H4	0.9800	C15—H15	0.9300
C5—C6	1.510 (3)	C16—C17	1.374 (4)
C5—C10	1.539 (4)	C17—C18	1.377 (4)
C5—H5	0.9800	C17—H17	0.9300
C6—C7	1.336 (3)	C18—H18	0.9300
C6—C12	1.499 (3)	C19—C20	1.383 (3)
C7—C19	1.476 (3)	C19—C24	1.385 (3)
C7—C8	1.521 (3)	C20—C21	1.376 (4)
C8—C9	1.543 (3)	C20—H20	0.9300
C8—H8	0.9800	C21—C22	1.363 (4)
C9—C10	1.538 (4)	C21—H21	0.9300
C9—H9A	0.9700	C22—C23	1.365 (4)
C9—H9B	0.9700	C23—C24	1.383 (3)
C10—H10A	0.9700	C23—H23	0.9300
C10—H10B	0.9700	C24—H24	0.9300
			
C2—C1—C11	135.9 (3)	C1—C11—H11A	109.5
C2—C1—C4	94.5 (2)	C1—C11—H11B	109.5
C11—C1—C4	129.5 (2)	H11A—C11—H11B	109.5
C1—C2—C13	136.3 (2)	C1—C11—H11C	109.5
C1—C2—C3	93.5 (2)	H11A—C11—H11C	109.5
C13—C2—C3	130.17 (19)	H11B—C11—H11C	109.5
C2—C3—C8	119.23 (18)	C6—C12—H12A	109.5
C2—C3—C4	85.80 (18)	C6—C12—H12B	109.5
C8—C3—C4	108.89 (17)	H12A—C12—H12B	109.5
C2—C3—H3	113.3	C6—C12—H12C	109.5
C8—C3—H3	113.3	H12A—C12—H12C	109.5
C4—C3—H3	113.3	H12B—C12—H12C	109.5
C1—C4—C5	118.5 (2)	C18—C13—C14	116.7 (2)
C1—C4—C3	86.23 (18)	C18—C13—C2	123.2 (2)
C5—C4—C3	109.0 (2)	C14—C13—C2	120.0 (2)
C1—C4—H4	113.4	C15—C14—C13	122.2 (2)
C5—C4—H4	113.4	C15—C14—H14	118.9
C3—C4—H4	113.4	C13—C14—H14	118.9
C6—C5—C10	108.8 (2)	C16—C15—C14	119.0 (2)
C6—C5—C4	109.20 (19)	C16—C15—H15	120.5
C10—C5—C4	106.8 (2)	C14—C15—H15	120.5
C6—C5—H5	110.6	C15—C16—C17	120.9 (2)
C10—C5—H5	110.6	C15—C16—Cl1	119.6 (2)
C4—C5—H5	110.6	C17—C16—Cl1	119.5 (2)
C7—C6—C12	126.8 (2)	C16—C17—C18	119.3 (2)
C7—C6—C5	113.71 (19)	C16—C17—H17	120.4
C12—C6—C5	119.4 (2)	C18—C17—H17	120.4
C6—C7—C19	126.06 (19)	C17—C18—C13	122.0 (2)
C6—C7—C8	113.95 (19)	C17—C18—H18	119.0
C19—C7—C8	119.98 (18)	C13—C18—H18	119.0
C7—C8—C3	108.55 (18)	C20—C19—C24	117.5 (2)
C7—C8—C9	108.40 (18)	C20—C19—C7	120.6 (2)
C3—C8—C9	107.24 (19)	C24—C19—C7	121.9 (2)
C7—C8—H8	110.8	C21—C20—C19	121.5 (2)
C3—C8—H8	110.8	C21—C20—H20	119.2
C9—C8—H8	110.8	C19—C20—H20	119.2
C10—C9—C8	109.4 (2)	C22—C21—C20	119.3 (3)
C10—C9—H9A	109.8	C22—C21—H21	120.3
C8—C9—H9A	109.8	C20—C21—H21	120.3
C10—C9—H9B	109.8	C21—C22—C23	121.0 (2)
C8—C9—H9B	109.8	C21—C22—Cl2	120.0 (2)
H9A—C9—H9B	108.3	C23—C22—Cl2	119.0 (2)
C9—C10—C5	109.39 (19)	C22—C23—C24	119.2 (2)
C9—C10—H10A	109.8	C22—C23—H23	120.4
C5—C10—H10A	109.8	C24—C23—H23	120.4
C9—C10—H10B	109.8	C23—C24—C19	121.2 (2)
C5—C10—H10B	109.8	C23—C24—H24	119.4
H10A—C10—H10B	108.2	C19—C24—H24	119.4
			
C11—C1—C2—C13	−1.7 (5)	C4—C3—C8—C9	61.1 (2)
C4—C1—C2—C13	−177.6 (3)	C7—C8—C9—C10	54.9 (3)
C11—C1—C2—C3	176.4 (3)	C3—C8—C9—C10	−62.1 (2)
C4—C1—C2—C3	0.50 (19)	C8—C9—C10—C5	0.1 (3)
C1—C2—C3—C8	−109.8 (2)	C6—C5—C10—C9	−55.6 (3)
C13—C2—C3—C8	68.5 (3)	C4—C5—C10—C9	62.2 (3)
C1—C2—C3—C4	−0.48 (18)	C1—C2—C13—C18	−9.6 (4)
C13—C2—C3—C4	177.8 (2)	C3—C2—C13—C18	172.8 (2)
C2—C1—C4—C5	109.1 (2)	C1—C2—C13—C14	171.1 (3)
C11—C1—C4—C5	−67.2 (3)	C3—C2—C13—C14	−6.5 (4)
C2—C1—C4—C3	−0.48 (18)	C18—C13—C14—C15	0.7 (4)
C11—C1—C4—C3	−176.8 (3)	C2—C13—C14—C15	−179.9 (2)
C2—C3—C4—C1	0.43 (16)	C13—C14—C15—C16	−1.5 (4)
C8—C3—C4—C1	119.93 (19)	C14—C15—C16—C17	1.1 (4)
C2—C3—C4—C5	−118.5 (2)	C14—C15—C16—Cl1	−176.6 (2)
C8—C3—C4—C5	1.0 (3)	C15—C16—C17—C18	0.1 (4)
C1—C4—C5—C6	−41.3 (3)	Cl1—C16—C17—C18	177.8 (2)
C3—C4—C5—C6	54.8 (3)	C16—C17—C18—C13	−0.9 (5)
C1—C4—C5—C10	−158.8 (2)	C14—C13—C18—C17	0.5 (4)
C3—C4—C5—C10	−62.6 (2)	C2—C13—C18—C17	−178.8 (3)
C10—C5—C6—C7	57.9 (3)	C6—C7—C19—C20	125.7 (3)
C4—C5—C6—C7	−58.3 (3)	C8—C7—C19—C20	−54.0 (3)
C10—C5—C6—C12	−120.4 (2)	C6—C7—C19—C24	−55.3 (3)
C4—C5—C6—C12	123.4 (2)	C8—C7—C19—C24	125.1 (2)
C12—C6—C7—C19	−1.3 (4)	C24—C19—C20—C21	3.9 (4)
C5—C6—C7—C19	−179.5 (2)	C7—C19—C20—C21	−177.0 (2)
C12—C6—C7—C8	178.3 (2)	C19—C20—C21—C22	−0.5 (4)
C5—C6—C7—C8	0.2 (3)	C20—C21—C22—C23	−3.1 (4)
C6—C7—C8—C3	58.3 (3)	C20—C21—C22—Cl2	177.3 (2)
C19—C7—C8—C3	−122.0 (2)	C21—C22—C23—C24	3.2 (4)
C6—C7—C8—C9	−57.9 (3)	Cl2—C22—C23—C24	−177.2 (2)
C19—C7—C8—C9	121.8 (2)	C22—C23—C24—C19	0.3 (4)
C2—C3—C8—C7	40.1 (3)	C20—C19—C24—C23	−3.7 (4)
C4—C3—C8—C7	−55.8 (2)	C7—C19—C24—C23	177.2 (2)
C2—C3—C8—C9	156.98 (19)		
